# Successful Treatment of Vulvar Lichen Sclerosus Using Microfiltered and Preconditioned Suspension of Autologous Adipose‐Derived Stem Cells With Photothermal Biomodulation: A Case Report

**DOI:** 10.1002/ccr3.71261

**Published:** 2025-10-15

**Authors:** Paolo Mezzana, Hernán Pinto

**Affiliations:** ^1^ Plastic Surgery Department Delle Medical Center Rome Italy; ^2^ Scientific Department Meta Cell Technology Barcelona Spain

**Keywords:** adipose‐derived stem cells, exosomes, photothermal biomodulation, vulvar lichen sclerosus

## Abstract

Vulvar lichen sclerosus is a chronic, inflammatory, autoimmune mucocutaneous disease characterized by hypopigmentation, skin sclerosis, and atrophy. Injections with microfiltered and preconditioned suspension of autologous adipose–derived stem cells are well tolerated and reduce symptoms and skin sclerosis without side effects or technical complications. Results are valid for 5 months after treatment (follow‐up period).

## 1 Introduction

Lichen sclerosus (LS) is a chronic, inflammatory, autoimmune mucocutaneous disease with a genetic component [1], with a predilection for female anogenital epithelium in 85%–98% of cases [2]. Other origins of LS, especially in males, include occlusion and irritation from irritant fluids such as urine [3, 4, 5]. Vulvar LS (VLS) is characterized by hypopigmentation, skin sclerosis, and atrophy that can lead to scarring, sexual dysfunction, and malignancy [6]. Advanced disease has a markedly deleterious impact on quality of life and is associated with an elevated risk of developing vulvar squamous cell carcinoma (SCC) [7].

VLS typically presents with itching, burning, and dyspareunia, although some patients remain asymptomatic. The most common signs for identifying the disease are hyperkeratosis and sclerosis, erosion, atrophy, erythema, and purpuric lesions and/or excoriations related to itching [8]. While the exact cause of VLS remains unclear, several factors contribute to its development. These include autoimmune factors (with most cases associated with other autoimmune diseases, such as thyroid disorders and vitiligo), hormonal imbalances, or genetics [1]. The differential diagnosis of VLS includes conditions such as thrush, postmenopausal atrophy, lichen planus, localized scleroderma, leukoplakia, and vitiligo, as well as immunobullous disorders such as cicatricial pemphigoid, cutaneous Lyme disease, and vulvar intraepithelial neoplasia (VIN) [9, 10, 11].

This case highlights a novel regenerative approach for treating VLS in a patient with autoimmune comorbidity, focusing on the use of microfiltered and preconditioned autologous adipose–derived stem cells (ADSCs).

## 2 Case Presentation/Examination

**FIGURE 1:**
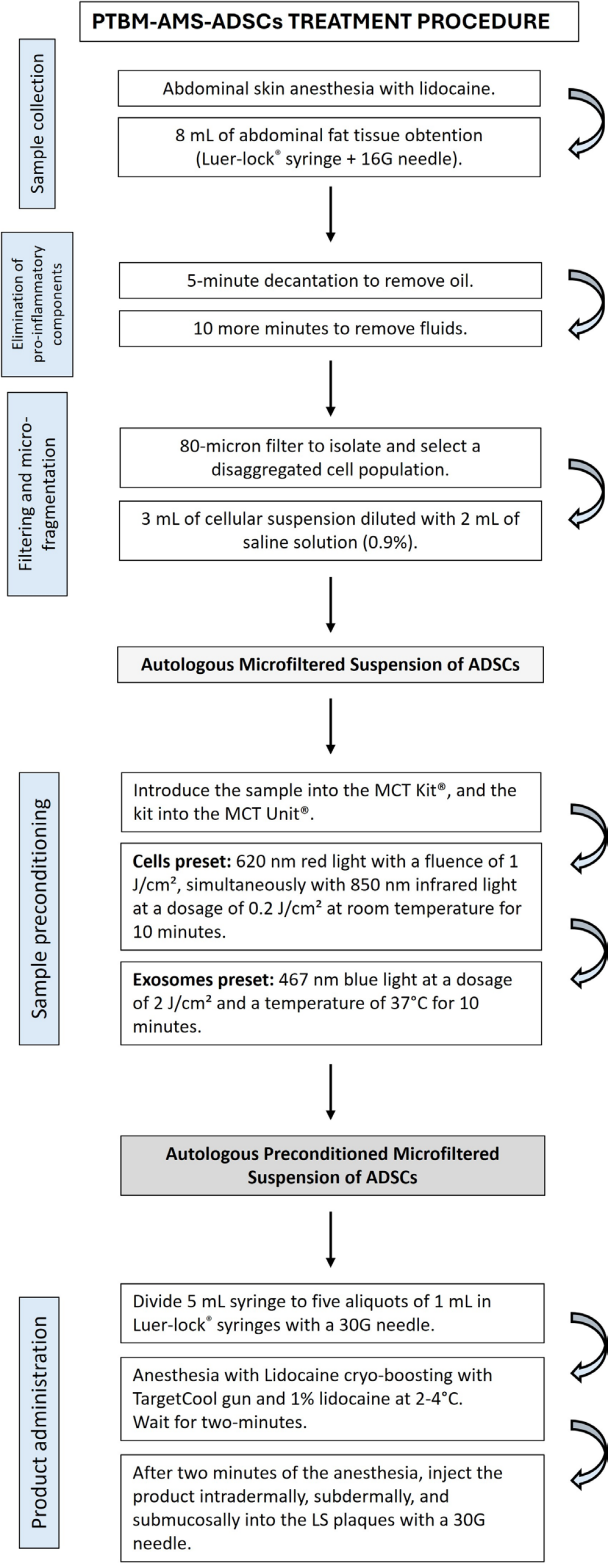
Microfiltered and preconditioning with photothermal biomodulation of a suspension of autologous adipose–derived stem cells treatment procedure. ADSC, adipose‐derived stem cells; LS, lichen schlerosus; PTBM‐AMS‐ADSCs, photothermal biomodulated autologous microfiltered suspension of adipose‐derived stem cells.

A 59‐year‐old woman with a history of Hashimoto thyroiditis was diagnosed with VLS in 2022. The patient mentioned a burning sensation and pain during intercourse, as well as dryness and persistent itching. These symptoms were consistent throughout the day, with no variation at night. The physical examination revealed the fusion of the labia minora and the presence of three white plaques, each measuring approximately 1 cm: two located at the labia minora and one at the vestibulum—all with signs of skin hyperkeratosis, sclerosis, and atrophy. The clinical diagnosis was established by consensus among three gynecologists and two dermatologists. A biopsy was not performed because there was no diagnostic uncertainty or suspicion of preneoplastic or neoplastic change [9]. Figure 1 summarizes all the procedures described below.

## 3 Methods (Differential Diagnosis, Investigations, and Treatment)

### 3.1 Previous Treatments

After explaining the standard treatment options, the patient refused treatment with glucocorticoids. Considering this, the patient was treated with local hyaluronic acid (HA) and autologous platelet‐rich plasma (PRP). The treatment regimen consisted of three sessions, with a 1‐month interval between sessions. While this treatment reduced itching, the plaques remained unchanged, and no adverse reactions were observed.

### 3.2 Treatment With Microfiltered and Preconditioned Suspension of Autologous ADSCs

The plaques were treated with a microfiltered and preconditioned suspension of autologous ADSCs obtained from abdominal adipose tissue. The microfiltered suspension was obtained with the MilliGraft kit (MilliGraft, Chieri, Italy). The preconditioning with photothermal biomodulation (PTBM) was performed using the MCT System (Meta Cell Technology, Sant Cugat del Vallés, Spain), which applies specific combinations of energy, wavelength, and duration to prime cells and promote the release of exosomes.

#### 3.2.1 Obtention of Microfiltered Suspension of Autologous ADSCs

The microfiltered suspension of autologous ADSCs was obtained from patients' adipose tissue following the MilliGraft procedure: (1) Regional anesthetic with 20% lidocaine ointment for vulgo‐vaginal desensitization and 5 mL of 2% lidocaine (Lidocaine Hydrochloride, Salf SpA, Bergamo, Italy) were diluted with 15 mL of saline solution 0.9%, and 0.1 mL of adrenaline for the harvest. (2) Abdominal fat tissue lipoaspirate was collected using a 16G needle connected to a 10‐mL Luer‐lock syringe to minimize mechanical stress on the adipocytes. (3) Excised fat tissue was processed to reduce adipose clusters gradually.

First, 8 mL of harvested tissue were decanted for 5 min to remove oil, and then for an additional 10 min to eliminate any further fluids. This procedure resulted in a liquid suspension without proinflammatory, oily, and hematological components. Using the classic Tonnard method [12], the lipoaspirate was microfragmented and filtered through an 80‐μm filter to isolate and select the disaggregated ADSCs population. The resulting product (3 mL of cellular suspension) was diluted with 2 mL of 0.9% saline solution.

#### 3.2.2 Preconditioning Procedure With PTBM

Five milliliters of the disaggregated and filtered lipoaspirate were introduced into the MCT Kit (Meta Cell Technology, Sant Cugat del Vallès, Spain), a 10‐mL capacity disposable cassette classified as an MDR medical device of class IIa. Subsequently, the kit was placed into the MCT Unit (Meta Cell Technology, Sant Cugat del Vallès, Spain) classified as an MDR medical device Class IIb. The sample preconditioning involved two of the three presets available on the device. Initially, the “Cells” preset was applied, comprising 620 nm red light with a fluence of 1 J/cm^2^, simultaneously with 850 nm infrared light at a dosage of 0.2 J/cm^2^ at room temperature for 10 min. Next, the “Exosomes” preset was applied, comprising 467 nm blue light at a dosage of 2 J/cm^2^ and a temperature of 37°C for 10 min. After completing the two PTBM procedures, the sample was extracted from the kit and placed into a 5‐mL syringe. Subsequently, this volume was transferred to five 1‐mL Luer‐lock syringes with a 30G needle for intradermal, subdermal, and submucosal injection.

#### 3.2.3 Product Injection

**FIGURE 2:**
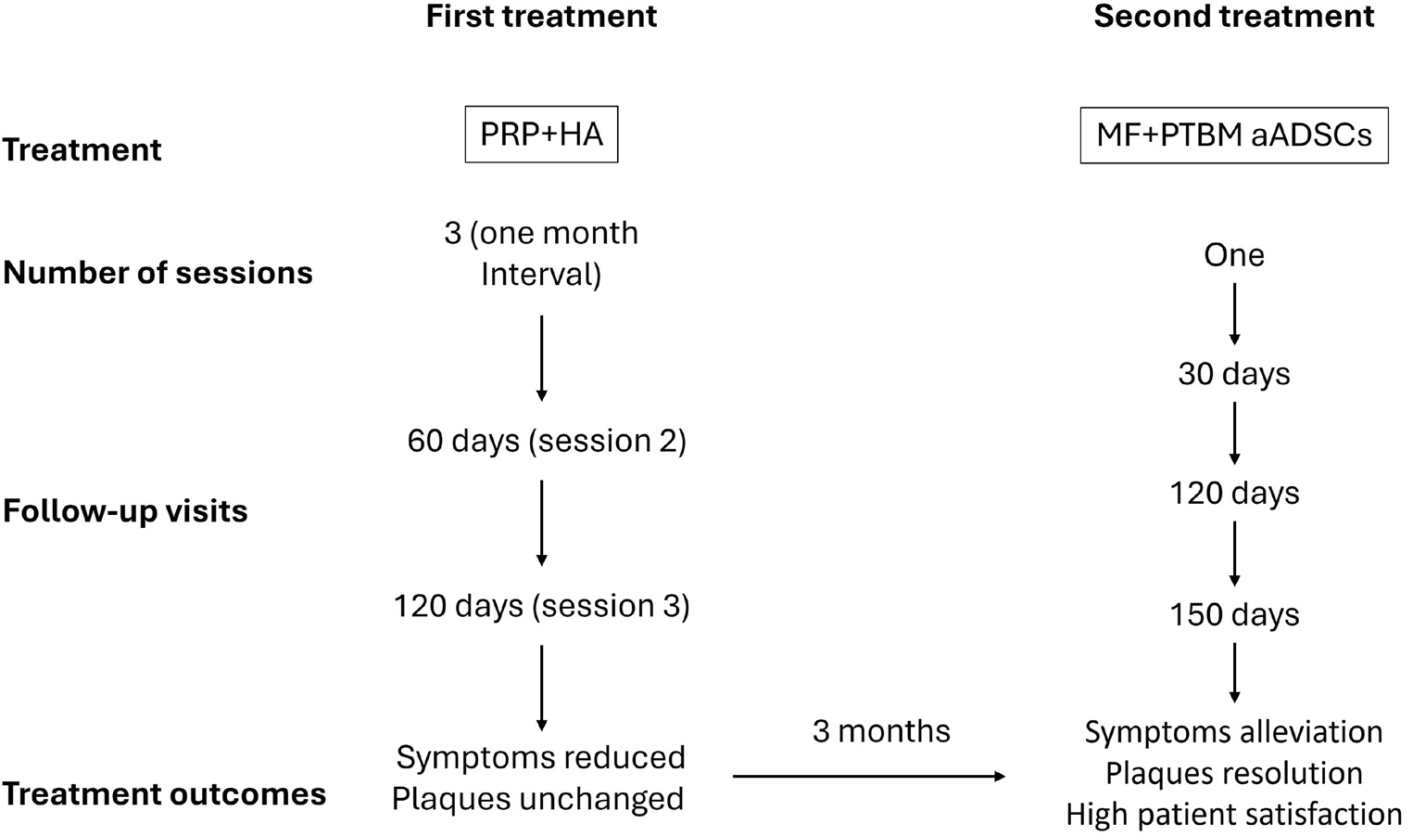
Timeline of treatments performed. HA, hyaluronic acid; MF+PTBM, microfiltered and photothermal biomodulated suspension of autologous adipose‐derived stem cells; PRP, platelet‐rich plasma.

Prior to treatment, a local anesthetic protocol comprising lidocaine (SALF SpA Italy, Bergamo, Italy)—referred to as lidocaine cryo‐boosting (LCB)—was applied on the affected area. This protocol was specifically designed for cell implantation procedures. Aerosolized 1% lidocaine at 2°C–4°C was administered using a TargetCool gun (CoolHealth, Anaheim, CA, USA) in boosting mode until the 2 mL reservoir was depleted. After 2 min, the microfiltered and PTBM‐preconditioned autologous ADSCs were injected intradermally, subdermally, and submucosally into the LS plaques using a 30G needle. The treatment regimen consisted of a single session with no additional treatment. Follow‐up visits were scheduled for 30, 120, and 150 days after treatment (Figure 2).

## 4 Conclusion and Results (Outcome and Follow‐Up)

The treatment with a microfiltered, PTBM‐preconditioned suspension of autologous ADSCs proved safe and effective, resulting in both symptom relief and marked reduction in skin sclerosis. The proposed mechanisms for applying this protocol were based on the anti‐inflammatory and regenerative properties of ADSCs, as well as the ability of preconditioning to increase exosome production and improve outcomes.

### 4.1 Treatment Outcomes and Safety

**FIGURE 3:**
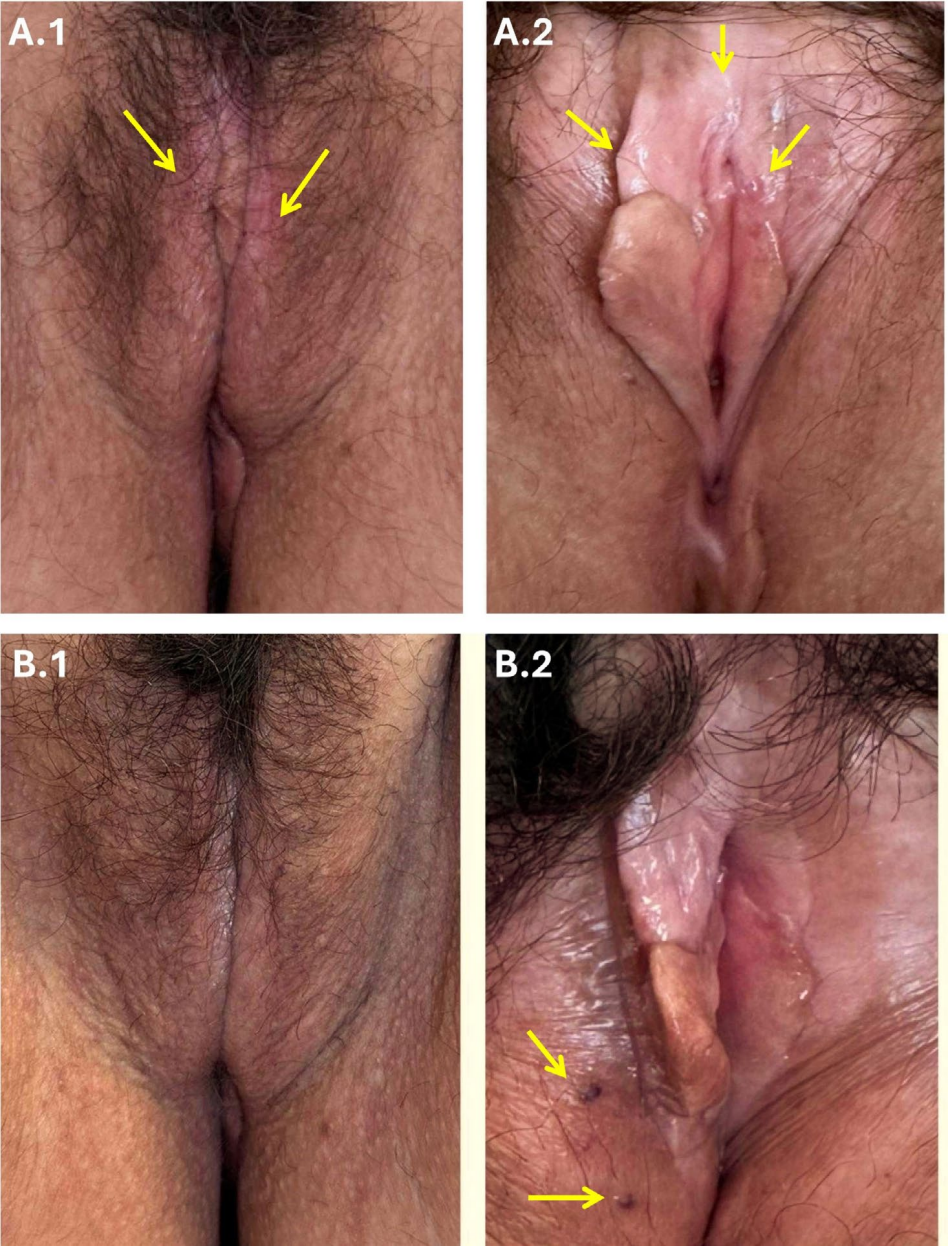
Photothermal biomodulated autologous microfiltered suspension of adipose‐derived stem cells treatment outcomes. (A1) Labia majora before treatment (yellow arrows mark white plaques observed at baseline with signs of skin hyperkeratosis or sclerosis and atrophy). (A2) Labia minora and vestibulum before treatment (yellow arrows mark white plaques observed at baseline with signs of skin hyperkeratosis or sclerosis and atrophy). (B1) Labia majora 5 months after treatment. (B2) Labia minora and vestibulum 5 months after treatment (yellow arrows mark transient treatment‐induced vascular dark spots, which are common after the treatment injections, require no medication, and resolve spontaneously).

At the follow‐up visits 30, 120, and 150 days after treatment, physical examination showed the resolution of plaques (Figure 3), alleviation of symptoms, and high patient satisfaction. The treatment was rapid and well tolerated. No severe side effects or complications were observed. Only two transient dark spots of vascular origin caused by the treatment appeared (Figure 3). Based on our experience, these spots can be found in some cases after treatment injections; they do not require medication and resolve without treatment. However, no published research articles were found to sustain this assertion.

## 5 Discussion

While numerous therapies exist to treat VLS, most offer limited regenerative potential. Ultrapotent topical corticosteroids remain the first‐line treatment and can alleviate symptoms; however, they rarely reverse structural damage, such as sclerosis, atrophy, and scarring—all of which contribute to the elevated risk of SCC [6, 13, 14, 15, 16]. Second‐line therapies with topical calcineurin inhibitors, such as pimecrolimus [17] or tacrolimus [18], have a cumulative risk of malignant transformation [19]. Estrogen, retinoids, various vitamins, phototherapy, surgical excision, and emollients are also recommended as supportive treatments [11, 20]. Surgical intervention is only indicated for treating complications associated with this condition [11, 21], but should be cautious due to the isomorphic phenomenon (Koebner) [22]. LS has been observed to exhibit occasional Koebnerization in response to stimuli such as radiotherapy [23] and physical trauma [24]. However, such occurrences can also occur associated with injection sites, but are rare and not typical [25].

In recent years, regenerative therapies such as PRP and ADSCs have shown promise in addressing the structural degeneration associated with VLS [26]. Both therapies improve VLS symptoms and possess significant anti‐inflammatory, angiogenic, and wound healing properties mediated through the secretion of cytokines, growth factors, and extracellular vesicles, particularly exosomes [27, 28, 29, 30, 31, 32]. Exosomes are lipid‐bilayer nanovesicles secreted by almost all cells between 30 nm and 150 nm in size [33], that positively affect recipient cells [34], by promoting cell proliferation and inhibiting apoptosis, mainly through exosomal miR‐323‐3p [35]. Evidence has confirmed that exosomes promote diabetic wound healing by inducing angiogenesis and collagen fiber deposition, while inhibiting inflammation [36, 37].

Photobiomodulation (PBM), or low‐level laser therapy, can precondition PRP or ADSCs by inducing cell proliferation and differentiation [38]. PBM has been shown to facilitate healing, reduce pain, and attenuate inflammation [39], by stimulating transcription factors that enhance cell viability, promote cell proliferation and migration, and induce protein production [39]. Some studies have reported that PBM increases angiogenesis markers while decreasing tissue maturation markers [40].

In the present case, PRP combined with HA provided partial symptomatic relief, consistent with PRP's known anti‐inflammatory capacity [41], and possibly enhanced by a synergistic interaction [42]. However, it failed to induce sustained tissue regeneration [42]. Symptoms recurred within 20 days after treatment, suggesting that the underlying pathology was not fully addressed. This fact led us to implement a novel protocol using microfiltered autologous ADSCs preconditioned with PTBM to induce exosome release and facilitate the restoration of normal tissue [43, 44].

The therapeutic rationale for applying this protocol was based on the anti‐inflammatory and regenerative capabilities of autologous ADSCs, as well as the effects of preconditioning to increase exosome release [45], and improve treatment outcomes. The microfiltration process removed oil, fat cell clusters, or fibrous tissue, which are present in the lipoaspirate [46]. These proinflammatory components can block the solution from passing through a 30G needle, impairing tissue restoration and leading to irreversible damage. Notably, preconditioning the microfiltered suspension of autologous ADSCs using the MCT System allowed rapid and complication‐free activation of ADSCs via PBM, enhancing their regenerative properties. Literature supports that ADSCs exert cytokine‐mediated paracrine effects that promote tissue regeneration [47], and secrete bioactive molecules, such as cytokines, antioxidants, chemokines, exosomes, and growth factors [48], promoting tissue regeneration and immunomodulation [49].

This advanced approach achieved complete symptom resolution and restoration of tissue integrity. Supporting evidence from other studies also demonstrates improved outcomes with fat grafting and ADSC‐based therapies in VLS. In nine studies and case reports using fat grafting as a treatment for VLS included in a literature review [50], patients reported improved symptoms, reduced fibrosis, and enhanced skin appearance [51, 52, 53, 54, 55, 56, 57, 58]. In a long‐term follow‐up study, 88.7% of patients treated for VLS were satisfied with fat grafting up to 11 years after surgery [59].

Additionally, the LCB used in this protocol, besides its primary anesthetic effect, promotes reactive vasodilation following the initial vasoconstriction, which supports effective cell implantation, as suggested by Lei et al. [60]. In vivo studies have found that a hypothermic microenvironment decreases lymphocyte proliferation, cytotoxic CD8^+^ T‐cell function, and the expression of interferon‐gamma (IFN‐γ) and IL‐2 [61].

Although the treatment showed favorable clinical outcomes, limitations include the absence of pre‐ and post‐treatment histological evaluation and a relatively short follow‐up period. Nonetheless, the data support this protocol as a safe and effective therapeutic alternative. Based on our results, our recommendation for future treatments would be one infiltration every 6 months to maintain remission.

## Author Contributions

**Paolo Mezzana:** conceptualization, data curation, formal analysis, investigation, methodology, project administration, resources, supervision, validation, visualization, writing – review and editing. **Hernán Pinto:** conceptualization, methodology, supervision, writing – original draft, writing – review and editing.

## Ethics Statement

The procedure followed the revised Declaration of Helsinki and Good Clinical Practice principles and complied with all applicable laws and regulatory requirements in Italy. Before any procedure, the patient signed an informed consent and gave her approval for the use and publication of their images. However, it was not reviewed by any Institutional Review Board.

## Consent

Written informed consent was obtained from the patient for the publication of this case and accompanying images.

## Conflicts of Interest

P.M. declares that Meta Cell Technology paid the article processing charges and provided material for the treatment, but he did not receive any personal fee for conducting the study. H.P. declares that he is an employee of Meta Cell Technology but did not receive any additional fee for his role in this study.

## Data Availability

The authors have nothing to report.
